# Optimizing Tomato Seed Performance Through Cold Atmospheric Plasma: Effects on Germination Rates and Early Biomass Development

**DOI:** 10.3390/plants15132093

**Published:** 2026-07-06

**Authors:** Adriana-Florica Bogoșel, Mihail Lungu, Oana-Alexandra Găinaru, Nicoleta Ianovici

**Affiliations:** 1 Faculty of Physics and Mathematics, Physics Department, West University of Timișoara, 300223 Timisoara, Romania; adriana.bogosel99@e-uvt.ro; 2 Faculty of Chemistry, Biology, Geography, Biology Department, West University of Timișoara, 300115 Timisoara, Romania; oana.gainaru@e-uvt.ro (O.-A.G.); nicoleta.ianovici@e-uvt.ro (N.I.); 3 Environmental Biology and Biomonitoring Research Center, The Institute for Advanced Environmental Research Timișoara, 300086 Timisoara, Romania

**Keywords:** atmospheric cold plasma (CAP), *Solanum lycopersicum* seeds, germination, fresh biomass, dry biomass, mineral biomass

## Abstract

Modern agriculture faces increasing pressure from rising food demand, resource degradation, and biotic stress factors, highlighting the need for sustainable, non-chemical technologies. Cold atmospheric plasma (CAP) has emerged as a promising non-chemical seed-priming technology with potential applications in sustainable agriculture. The present study investigated the effects of dielectric barrier discharge (DBD)-generated CAP on seed germination and early seedling development in two *Solanum lycopersicum* genotypes (a common variety and an IdB hybrid) under controlled laboratory conditions. Seeds were exposed to CAP for 1, 2, 3, or 4 min, while untreated seeds served as controls. Early plant performance was evaluated after 47 days by determining germination rate, fresh biomass, dry biomass, and mineral biomass (ash content). CAP exposure duration significantly affected all gravimetric parameters in both genotypes. Among the tested treatments, 1 min exposure consistently produced the highest fresh, dry, and mineral biomass values, whereas longer exposure times (3–4 min) generally reduced seedling growth, indicating the transition from beneficial physiological stimulation to stress-induced inhibition. Despite the more pronounced response observed in the IdB hybrid, the statistical analysis demonstrated that treatment duration, rather than genotype, was the principal factor influencing biomass accumulation. The present results indicate that short-duration CAP treatment represents an effective seed-priming strategy for improving early tomato seedling development.

## 1. Introduction

Nowadays, agriculture is confronted with the challenge of increasing food production while simultaneously reducing environmental impact and preserving natural resources. According to the United Nations Sustainable Development Goals (SDGs), future agricultural systems must contribute to achieving Zero Hunger (SDG 2) while ensuring responsible production practices (SDG 12), climate resilience (SDG 13) and the protection of terrestrial ecosystems (SDG 15) [1,2]. However, the intensification of agricultural practices during recent decades has relied heavily on synthetic fertilizers and pesticides, resulting in soil degradation, water contamination, biodiversity loss and the emergence of resistant pest populations [3,4,5]. Consequently, the development of sustainable technologies capable of improving crop productivity while reducing dependence on chemical inputs has become a major scientific and societal priority. These objectives are further reflected in the European Green Deal [6] and the Farm to Fork Strategy [7], which promote a substantial reduction in pesticide use and encourages the adoption of environmentally friendly technologies throughout the agricultural sector.

Among the various approaches developed to improve crop establishment and plant performance, seed priming technologies have attracted considerable scientific interest. Conventional priming methods, including hydropriming, osmopriming, chemical priming, and microbial inoculation have demonstrated positive effects on germination and seedling development [8,9,10,11]. However, their efficiency may be influenced by environmental conditions, storage limitations, treatment reproducibility, and, in some cases, the use of chemical compounds [12]. Therefore, increasing attention has been directed toward physical seed-treatment technologies that can induce beneficial physiological responses without introducing additional chemical substances into agricultural systems.

Cold atmospheric plasma (CAP) has emerged as one of the most promising non-chemical technologies for agricultural applications [13,14]. Plasma is commonly described as the fourth state of matter and consists of partially ionized gas containing electrons, ions, radicals, ultraviolet photons, and excited atoms and molecules [15]. Unlike thermal plasmas, CAP operates at temperatures close to ambient conditions, allowing direct treatment of biological materials without causing thermal damage [15].

Several plasma-generation systems have been investigated for agricultural purposes, including corona discharges, plasma jets, microwave plasmas, radio-frequency plasmas, gliding arc discharges, and dielectric barrier discharges (DBD) [16,17]. Among these technologies, DBD systems have received particular attention because they can generate stable plasma under atmospheric-pressure conditions without requiring expensive vacuum equipment. Furthermore, DBD reactors allow the treatment of relatively large sample surfaces, provide homogeneous exposure of seed batches, and can be scaled for industrial implementation [18]. These characteristics make DBD one of the most practical and economically feasible plasma-generation methods for agricultural applications.

In dielectric barrier discharge systems, plasma is generated between two electrodes separated by at least one dielectric material, typically consisting of glass, quartz, or ceramic [19]. The dielectric barrier limits electrical current and prevents arc formation, resulting in the generation of numerous transient micro-discharges distributed throughout the discharge gap [20]. These micro-discharges produce a complex mixture of reactive oxygen species (ROS) and reactive nitrogen species (RNS), including ozone ($O_{3}$), hydroxyl radicals (${OH}^{•}$), hydrogen peroxide ($H_{2}O_{2}$), nitric oxide (NO), and nitrogen oxides (NO_x_), which are considered the primary mediators of cold atmospheric plasma-induced biological effects [21].

The interaction of cold atmospheric plasma-generated reactive species with the seed surface can induce significant physicochemical modifications, including increased wettability, enhanced water absorption, microbial decontamination, and activation of metabolic pathways associated with germination and plant growth [22]. Moreover, CAP treatment has been reported to stimulate antioxidant defense mechanisms, regulate phytohormonal signaling pathways, and improve plant tolerance to abiotic and biotic stress conditions [23]. As a result, plasma agriculture has emerged as a rapidly expanding research field with potential applications in sustainable crop production and integrated pest management [24].

Tomato (*Solanum lycopersicum*) was selected as the experimental model species due to its exceptional agricultural, economic, and scientific importance. Tomato is one of the most widely cultivated vegetable crops worldwide, with annual production reaching 188.5 million tons and a harvested area of over 5.1 million hectares in 2024 [25]. In addition to its economic relevance, tomato has become one of the most extensively studied model plants in modern plant science owing to its relatively short life cycle, diploid genome, extensive genomic resources [26] and well-established transformation protocol [27]. Consequently, tomato has been widely employed in studies addressing plant physiology, molecular genetics, stress biology, host–pathogen interactions and crop improvement strategies [28,29,30,31,32,33].

The selection of tomato is further justified by its prominent role in plasma-agriculture research. Numerous studies have demonstrated that plasma treatment of tomato seeds can improve germination percentage, accelerate seedling emergence, increase root and shoot growth, modify seed coat morphology, enhance antioxidant activity and influence the expression of genes involved in stress responses and plant development [34,35,36,37,38,39].

The present study aims to investigate the effects of CAP treatment on seeds of two *Solanum lycopersicum* varieties and to determine its impact on germination and early seedling growth under controlled laboratory conditions. Seedling performance was assessed through biomass-related parameters, based on which the contribution of genetic background to the variability of biological responses induced by CAP exposure was simultaneously evaluated.

## 2. Results

### 2.1. CAP Treatment Influence on Germination and Early Seedling Development

Two *Solanum lycopersicum* genotypes were evaluated under controlled laboratory conditions in this study: a common variety and an IdB hybrid. After a period of 47 days, the germination rate of treated seeds yielded the following results:

- *Solanum lycopersicum* common variety: 42 seedlings (overall germination rate 42%), distributed as 8 samples in the 1 min treatment lot (40% germination rate), 9 samples in the 2 min treatment lot (45% germination rate), 6 samples in the 3 min treatment lot (30% germination rate), 9 samples in the 4 min treatment lot (45% germination rate) and 10 samples in the control lot (50% germination rate);
- *Solanum lycopersicum* IdB hybrid: 49 seedlings (overall germination rate 49%), distributed as 11 samples in the 1 min treatment lot (55% germination rate), 4 samples in the 2 min treatment lot (20% germination rate), 12 samples in the 3 min treatment lot (60% germination rate), 9 samples in the 4 min treatment lot (45% germination rate) and 13 samples in the control lot (65% germination rate).

Following germination, development of seedlings obtained from cold atmospheric plasma-treated seeds was carried out by determining the fresh and dry biomass, and ash content for each experimental variant (Table 1).

**Figure 1:**
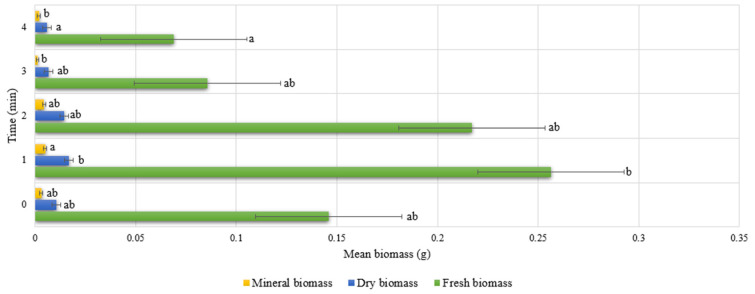
Biomass variation of the common variety of *Solanum lycopersicum* under different treatment times. Values are presented as mean ± SD. Different lowercase letters indicate statistically significant differences among treatment times within each biomass type according to Tukey’s HSD test (fresh and dry biomass) and Dunnett’s test (ash content) (*p* < 0.05). Bars sharing at least one lowercase letter are not significantly different, whereas bars with no lowercase letters in common differ significantly.

**Figure 2:**
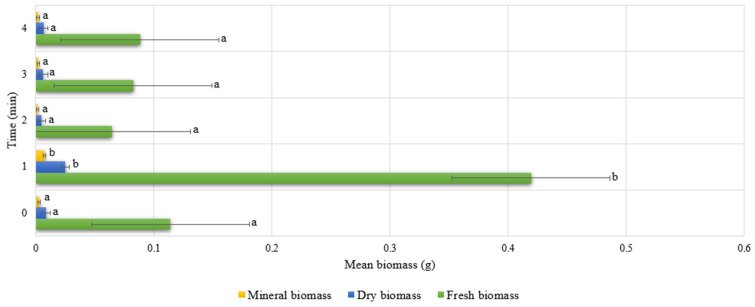
Biomass variation of the *Solanum lycopersicum* IdB hybrid under different treatment times. Values are presented as mean ± SD. Different lowercase letters indicate statistically significant differences among treatment times within each biomass type according to Tukey’s HSD test (*p* < 0.05). Bars sharing at least one lowercase letter are not significantly different, whereas bars with no lowercase letters in common differ significantly.

The variations of mean biomass values according to CAP treatment time are presented in Figure 1—for *Solanum lycopersicum* common variety and Figure 2—for *Solanum lycopersicum* IdB hybrid.

The mean values of fresh biomass showed a dependence on CAP exposure duration in both tomato genotypes (Table 1). The highest mean values were recorded following the 1 min treatment, for both the common variety and the IdB hybrid of *Solanum lycopersicum*. The scale of the response was more marked in the IdB hybrid, which showed a higher biomass value than the corresponding control group. In contrast, treatments exceeding 1 min were associated with gradually decreased fresh biomass values.

A similar trend was observed for mean values of dry biomass (Table 1). For both genotypes, the maximum mean value for dry biomass was obtained after 1 min of CAP treatment, whereas longer exposure duration resulted in lower values.

Also, the mean values of mineral biomass (ash content) exhibited a time-dependent response. In the common variety of *Solanum lycopersicum*, the highest values were recorded after 1–2 min of CAP exposure, whereas in the IdB hybrid—the maximum was observed after 1 min of treatment. For both genotypes, exposure duration of 3–4 min were generally associated with reduced mean values of mineral biomass.

Normality of biomass data was assessed using the Shapiro–Wilk test for each experimental group. The results showed no significant deviation from normality in any group (control: *p* = 0.150; 1 min: *p* = 0.641; 2 min: *p* = 0.193; 3 min: *p* = 0.529; 4 min: *p* = 0.277), with all *p*-values exceeding the significance threshold of 0.05.

#### 2.1.1. Analysis of the Effect of Cold Plasma Treatment on *Solanum lycopersicum* Common Variety

The evaluation of the impact of different cold plasma exposure durations on the fresh biomass of *Solanum lycopersicum* common variety showed that the best results were obtained at the 1 min exposure, with an average of 0.2565 g, while the lowest biomass was observed for the 4 min treatment, at 0.0688 g (Table 1). The values for the other durations were intermediate: 0.2171 g for 2 min, 0.0858 g for 3 min, while the untreated control showed an average of 0.1459 g. To test whether these differences between groups are significant, a classic ANOVA test was applied (Table 2). The results showed a significant global difference between groups (F = 3.933, *p* = 0.0095), with a large effect size (omega^2^ = 0.2225).

**Figure 3:**
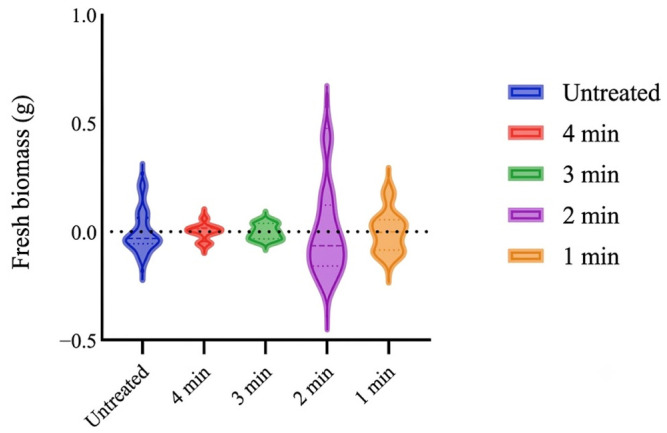
Violon plot—fresh biomass of *Solanum lycopersicum* common variety.

The violin plot representation (Figure 3) highlights relevant differences in the distribution of fresh biomass values based on the exposure time of *Solanum lycopersicum* common variety seeds to cold atmospheric plasma treatment. The 2 min treatment shows the greatest vertical extent of the distribution, indicating high variability in the biological response and a wide range of individual values. This dispersion suggests a heterogeneous effect of exposure, where some plants show increased values, while others record decreases in biomass. Meanwhile, the 1 min treatment is characterized by a more compact distribution, concentrated in a range moderately higher than the control, indicating a relatively stable and consistent response among replicates. In contrast, the 4 min treatment shows a shift in value density toward the lower end of the axis, suggesting a general reduction in fresh biomass. The 3 min group shows considerable overlap with the control, reflecting a neutral or weakly expressed effect.

Furthermore, the variability between groups was moderate (ICC = 0.264). However, Levene’s test for homogeneity of variances yielded contradictory results: *p* = 0.0055 for mean variables (indicating unequal variances) and *p* = 0.0679 for medians (borderline acceptable). Given these results, Welch’s test was also employed (Table 2) to correct for the effects of unequal variances, confirming significant global differences between treatments (F = 7.607, *p* = 0.001041).

The Tukey HSD post-hoc analysis allowed for the precise identification of groups that differ statistically, the only significant difference being found between the 4 min and 1 min treatments, with *p* = 0.0251. The remaining comparisons, including those between the control and the 2 min and 3 min plasma treatments, did not show significant differences.

The biological interpretation of these results may suggest that the 1 min duration is optimal for maintaining fresh biomass, as its values are the highest and do not differ negatively from the control. In contrast, the prolonged 4 min exposure significantly decreases biomass, indicating the onset of physiological stress and water loss within the tissues. The intermediate durations of 2 and 3 min plasma treatments have a neutral or slightly negative effect on biomass and are not statistically significant compared to the control.

Regarding the dry biomass values, the ANOVA test results showed significant differences between treatments (F = 3.524, *p* = 0.0158), indicating that the duration of plasma exposure significantly influences dry biomass (Table 2). The treatment effect (omega^2^ = 0.1976) is moderate, and the ICC = 0.236 suggests a moderate variation between groups.

**Figure 4:**
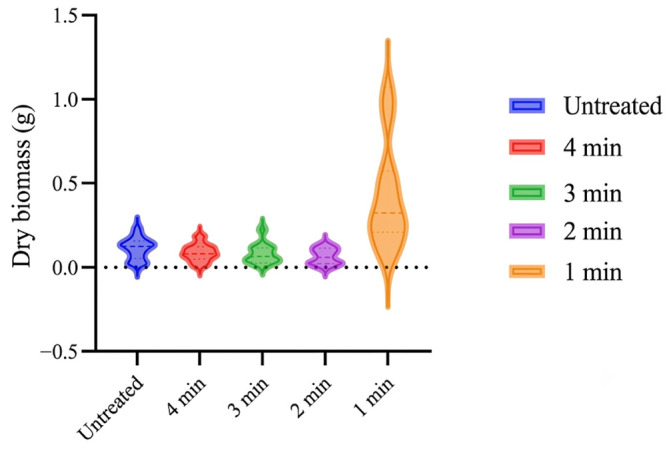
Violin plot—dry biomass of *Solanum lycopersicum* common variety.

The violin plot depicting the dry biomass of *Solanum lycopersicum* common variety seeds (Figure 4) subjected to CAP treatment reveals a distinct divergence in the 1 min exposure group. This group exhibits a distribution shifted towards higher values with negligible overlap relative to the other treatment cohorts. Conversely, the 2–4 min treatment intervals are characterized by concentrated distributions and significantly attenuated values.

The components of variance were also examined: the inter-group variability (Var(group) = 1.6 × 10^−5^) was found to be lower than the intra-group variability (Var(error) = 5.18 × 10^−5^). This pattern is typical of biological experiments, where inherent natural variation often exceeds treatment-induced differences. Given these findings, Welch’s ANOVA (Table 2) was performed to account for unequal variances (heteroscedasticity). The test confirmed significant global differences between treatments (F = 7.07, *p* = 0.00145).

The Tukey HSD post-hoc test revealed that the only statistically significant difference occurred between the 4 min treatment group and the 1 min treatment group, with *p* = 0.0251. All other pairwise comparisons remained non-significant, reflecting the pattern previously observed for fresh biomass.

From a biological perspective, the results suggest that a 1 min exposure to cold plasma could maintain the dry biomass at high levels without inducing physiological stress, whereas the 4 min exposure significantly reduces dry biomass, indicating adverse effects on the tissues. Intermediate treatments (2–3 min) did not elicit significant differences and may be considered acceptable options.

The ANOVA Test results regarding the mineral biomass (ash content) of the treated common tomato seeds demonstrated significant global differences between treatments (F = 2.753, *p* = 0.0423), indicating that the duration of plasma exposure can influence the mineral content of the plants (Table 2). The effect size (omega^2^ = 0.143) was moderate, and the ICC = 0.174 suggests that the inter-group variation is lower compared to the intra-group variation, a phenomenon typical of biological experiments.

**Figure 5:**
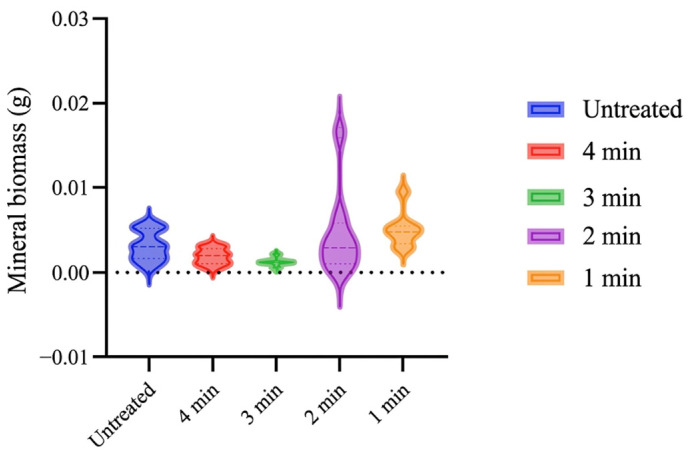
Violin plot—mineral biomass of *Solanum lycopersicum* common variety.

The violin plot for mineral biomass highlights more subtle differences compared to those observed for fresh and dry biomass. In Figure 5, it is noteworthy that the 2 min treatment exhibits the most extended and elongated violin shape, reflecting a significant dispersion of values. This suggests a heterogeneous response to this exposure duration, with some plants accumulating higher mineral content while others show a decrease in mineral biomass. In contrast, the 1 min treatment produces a more compact distribution concentrated at the upper end of the value scale, indicating a more stable and uniform response, with slightly increased mineral content compared to both the control and the longer treatment durations. This consistency suggests that short plasma exposure stimulates physiological processes favorable to mineral absorption and retention without inducing significant metabolic stress. The control and the 3 and 4 min treatments show narrower distributions with relatively low mean values, which may reflect reduced mineral accumulation or an impairment of the physiological mechanisms for mineral transport and storage. A distinct feature of the 3 min treatment is its narrow violin shape, signaling a homogeneous response; however, the values remain near zero, suggesting a neutral or slightly negative effect on mineral biomass.

The assumption of homogeneity of variances was verified using Levene’s test, which yielded mixed results: *p* = 0.0239 based on means (indicating slight differences in variance) and *p* = 0.1258 based on medians (suggesting variances are acceptably equal). To account for the potential effects of unequal variances, Welch’s ANOVA Test (Table 2) confirmed significant global differences between groups (F = 7.942, *p* = 0.0007271).

The observations derived from the violin plot are further supported by the fact that the Tukey HSD test identified no significant pairwise comparisons. Consequently, a complementary analytical method was employed—the Dunnett T3 multiple comparisons post-hoc test—which highlighted several differences, the mean ash content measured after 1 min of treatment being significantly higher compared to the 3 min treatment (adjusted *p* = 0.0217) and the 4 min treatment (adjusted *p* = 0.0482). This indicates that extending the treatment duration beyond 1 min leads to a statistically significant reduction in ash content. In contrast, no statistically significant differences were observed between the 1 min and 2 min treatments (adjusted *p* = 0.9699), suggesting that ash content remains comparable between these two exposure times. Similarly, the ash content at 1 min did not differ significantly from the control group (adjusted *p* = 0.3271), indicating that a 1 min treatment does not produce a statistically detectable change relative to untreated samples.

Overall, these results demonstrate that shorter exposure (1–2 min) maintains higher ash content values, whereas longer exposure times (3–4 min) are associated with a significant decrease in mineral deposition.

Consequently, the integrated analysis of mineral biomass—utilizing ANOVA, Welch’s test, and Dunnett’s test—indicates that plasma exposure duration influences mineral content, though the effects are more subtle than those observed for fresh or dry biomass. Dunnett’s test was employed for identifying specific inter-group differences, providing a more detailed perspective on the treatments’ impact.

#### 2.1.2. Analysis of the Effect of Cold Plasma Treatment on *Solanum lycopersicum* IdB Hybrid

The mean values for fresh biomass indicate that the 1 min treatment yielded the highest value, being considerably higher than all other exposure time lots (Table 1). The 2, 3, and 4 min treatments showed considerably lower values, while the control remained at an intermediate level, though almost four times lower than the optimal treatment.

To evaluate the significance of the differences between groups, a standard ANOVA test was performed (Table 3), which indicated a highly significant global difference (F = 9.233, *p* = 1.66 · 10^−5^).

**Figure 6:**
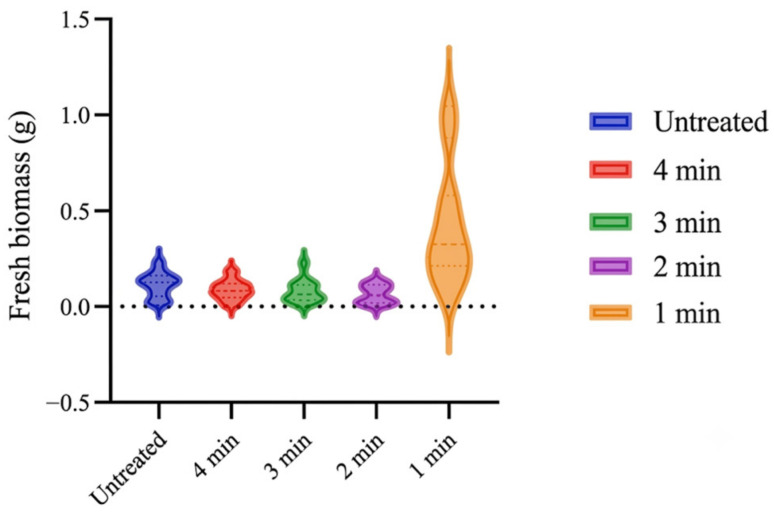
Violin plot—fresh biomass of *Solanum lycopersicum* IdB hybrid.

The distribution analysis of fresh biomass (violin plot) highlights the influence of CAP exposure duration on the hydration capacity and early growth of seedlings derived from *Solanum lycopersicum* IdB hybrid seeds (Figure 6). Compared to the control (untreated) variant, the 1 min treatment results in a visible increase in fresh biomass values, suggesting stimulation of water uptake and an activation of the metabolic processes involved in germination and early development. The 2, 3 and 4 min treatment variants show no significant improvements and indicate a tendency toward stagnation or reduction in values, possibly due to the onset of physiological stress induced by prolonged exposure. Welch’s ANOVA Test results (Table 3) confirmed the above conclusions (F = 3.349, *p* = 0.03579).

The Tukey HSD post-hoc analysis indicated that the only significant differences were between the 1 min treatment and all other groups. Longer exposure durations (3–4 min) showed highly significant differences compared to the 1 min interval, while the differences between the other durations (2, 3 and 4 min) and the control group did not reach the threshold of statistical significance.

Biological interpretation suggests that 1 min plasma treatment represents the optimal duration for maintaining and stimulating fresh biomass in *Solanum lycopersicum* IdB hybrid. Exposures exceeding 2 min induce notable decreases in biomass, suggesting a rapid onset of physiological stress associated with prolonged interaction with CAP generated reactive species.

The dry biomass analysis for IdB tomatoes confirms the pattern previously observed for fresh biomass, showing a highly sensitive response to exposure duration. The ANOVA Test results (Table 3) revealed significant global differences between groups (F = 8.447, *p* = 3.80 ∙ 10^−5^), with an omega^2^ = 0.3781 that indicates a large treatment effect.

**Figure 7:**
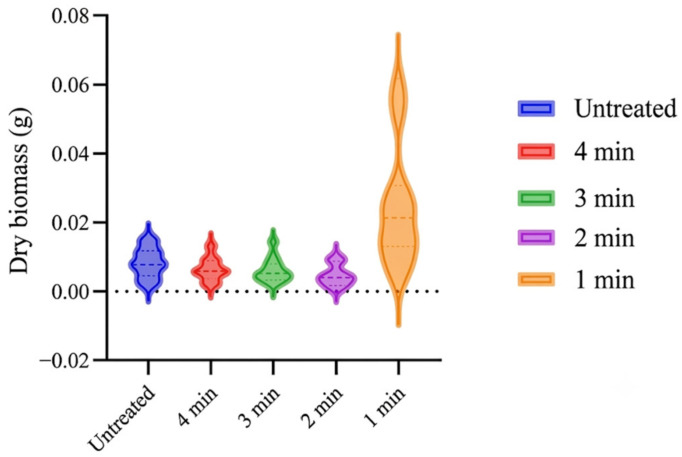
Violin plot—dry biomass of *Solanum lycopersicum* IdB hybrid.

In the case of dry biomass, which reflects the accumulation of organic matter resulting from synthesis and structural growth processes, the 1 min treatment presented the highest recorded values (Figure 7). This increase suggests effective stimulation of cell division and plant anabolic metabolism without producing harmful effects on cellular integrity. Longer exposure times (2–4 min) did not indicate the same level of efficiency, suggesting that an excessive dose of plasma-generated reactive species could begin to negatively impact normal physiological processes. Furthermore, Welch’s test (Table 3) confirmed these differences (F = 3.325, *p* = 0.03691).

The Tukey HSD post-hoc test shows that the 1 min treatment differs significantly from all other exposure durations. Biologically, these results may indicate that the 1 min treatment stimulates physiological processes and maintains an optimal cellular balance. In contrast, the 3 min and 4 min exposures could impair primary metabolism, leading to a decrease in dry biomass.

The evaluation of ash content in IdB hybrid implied a distinct behavior, both compared to its own fresh and dry biomass values and to the reaction previously observed in the common variety. Statistical analysis via ANOVA Test (Table 3) indicates the existence of significant differences between treatments (F = 7.518, *p* = 0.0001049), supported by a large effect size (omega^2^ = 0.3473), which confirms that the duration of exposure to cold atmospheric plasma influences the mineral content of the IdB hybrid.

**Figure 8:**
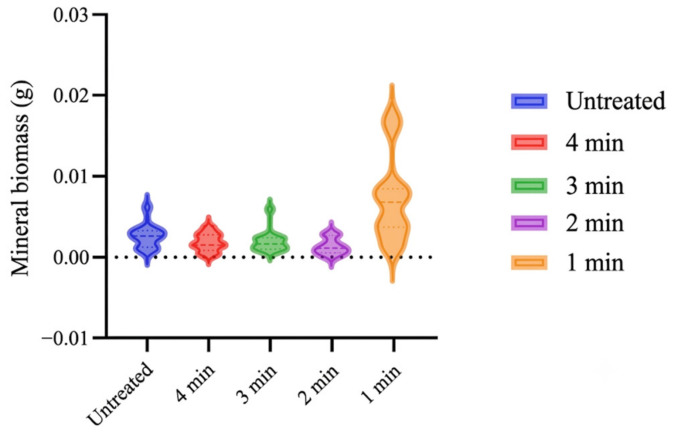
Violin plot—mineral biomass of *Solanum lycopersicum* IdB hybrid.

The higher values observed in the violin plot (Figure 8) suggest that short-term exposure to cold plasma improves physiological processes involved in the mechanisms of accumulation and retention of mineral substances. Conversely, longer treatments do not exhibit the same benefits, indicating a possible limitation or even inhibition of mineral absorption under conditions of excessive exposure. The Welch test (Table 3) results validates the observations (F = 3.168, *p* = 0.04229), even in the absence of homogeneity of variances.

The Tukey post-hoc analysis detected clear significant differences, showing that the 1 min treatment yields the highest mineral biomass values and is statistically distinct from all other durations. This disparity between tested groups could suggest an increased sensitivity of the IdB hybrid to cold plasma treatment parameters.

The biological interpretation of these results suggests that IdB tomatoes possess a pronounced capacity for mineral absorption and retention under conditions of short plasma exposure. The 1 min duration appears to stimulate physiological processes that optimize ion transport and cell membrane activity. Conversely, prolonged exposures (3 and 4 min) could generate a disruption of ionic homeostasis, likely by compromising membrane integrity or transmembrane transport mechanisms, leading to a significant decrease in mineral content.

### 2.2. Genetic Background Influence on Biological Response

A two-way ANOVA comparison was made in order to assess the possible differences between the two tested genotypes, taking into consideration the variety and treatment time as independent variables and the gravimetric parameters as the dependent one. The overall results are presented in Table 4.

The results obtained demonstrated a statistically insignificant relationship regarding the two genotypes tested (common tomato variety and IdB hybrid), with it being concluded that the genetic variety of the studied plants did not influence the gravimetric results obtained. Moreover, the interaction between genotype and CAP exposure time did not present significant results, with CAP exposure time remaining the only variable that influenced plant growth and development, with large effect sizes in each of the three tested instances (ω^2^ being 0.3257 for fresh biomass, 0.3002 for dry biomass, and 0.2520 for mineral biomass, respectively).

## 3. Discussion

Short exposure times of cold atmospheric plasma (CAP) have been consistently reported to enhance seed germination, seedling vigor, and early plant growth across multiple species, including tomato, soybean, wheat, and cotton [40,41,42,43,44,45]. This response pattern can be interpreted through a treatment time–response model, in which moderate exposure levels stimulate signaling pathways involved in cellular metabolism, antioxidant protection, and early developmental processes, whereas prolonged exposure may exceed the adaptive capacity of tissues, resulting in physiological stress and growth inhibition [46]. In this context, CAP treatment can be regarded as a controlled abiotic stimulus that resembles natural stress priming, thereby promoting plant resilience and adaptive responses during the early stages of ontogeny [44]. An optimal dose, at which seed germination is stimulated and no adverse effects could be noted on juvenile plants, has been determined for a variety of species, such as *Brassica napus* [47], *Hordeum vulgare* [48], *Lycopersicum esculentum* [49], *Andrographis paniculata* [50], *Arabidopsis thaliana* [51], *Oryza sativa* [52], *Glycine max* [53], *Papaver somniferum* [54], *Pinus nigra* [55], *Cucumis sativus* and *Capsicum annuum* [56], and *Triticum aestivum* [57,58].

In the present study, the integrated statistical approach—combining ANOVA, Welch’s correction, and Tukey’s post-hoc analysis—demonstrated that a short exposure duration of 1 min represents the optimal treatment for fresh biomass accumulation between the four evaluated treatments. This result is in accordance with previous reports showing that CAP seed treatments can significantly improve plant growth and productivity by modulating physiological activation processes rather than inducing tissue damage [49,59,60]. Mechanistically, this suggests that short cold atmospheric plasma exposure may enhance membrane permeability, stimulate water uptake, and activate metabolic enzymes involved in germination and biomass formation [46,48,61].

In contrast, longer exposure duration resulted in a clear decline in biomass, which could be interpreted as the transition from a favorable zone to a stress-inducing regime [62]. Such responses have been associated in the scientific literature with excessive accumulation of reactive species, leading to oxidative imbalance, lipid peroxidation and disruption of cellular homeostasis [48,57]. This dual behavior of CAP exposure closely mirrors dose-dependent physiological responses observed in plant stress biology, where low-level stress enhances tolerance, whereas high-intensity stress suppresses growth [63].

Similar patterns were observed for the dry biomass, where the 1 min treatment consistently produced statistically significant results. From a physiological perspective, dry biomass reflects the net accumulation of structural organic compounds, suggesting that short CAP exposure has the potential to enhance water uptake and stimulate carbon assimilation and biosynthetic activity. The present results align with findings reported in tomato and soybean experimental designs, where plasma treatments were shown to activate metabolic and growth-related pathways, leading to improved seedling development and yield potential [49,50].

The mineral biomass response exhibited a moderate and less pronounced pattern compared to fresh and dry biomass, suggesting that mineral uptake and accumulation processes are less sensitive to short-term plasma-induced signaling or may require longer exposure thresholds to manifest significant changes. Similar observations have been reported in previous studies, where biochemical and phytochemical modifications induced by plasma were present but less pronounced than morphological responses during early growth stages [52]. The current results support the hypotheses that CAP exposure primarily affects physiological activation and growth regulation rather than directly altering mineral homeostasis at short exposure durations.

Comparative tests indicated non-significant genotype-dependent results, the discrepancies between the IdB hybrid physiological responses and those of the common tomato variety potentially being attributed to the high variability within the treated lots. Contrary to the present findings, cultivar-dependent effects have been reported in tomato and other species, where plasma-induced responses varied significantly depending on genetic background and physiological state of the seed, reflecting the genetic variability in stress perception, antioxidant capacity, and metabolic regulation [41]. Furthermore, recent studies suggest that CAP may influence gene expression and epigenetic regulation pathways associated with energy metabolism and stress adaptation, which could explain the enhanced responsiveness observed in certain genotypes [51,53,64]. Plasma-induced seed surface modifications, as previously described in seed coat studies, may also contribute to improved water uptake and faster activation of germination-related pathways, underlining the importance of genotype-specific optimization when applying CAP as a seed priming technology [54,55].

The results obtained should also be interpreted in light of the inherent biological limitations associated with living organisms. Variability in the observed responses may be influenced by several factors, including seed coat thickness, the duration of dormancy release, and sensitivity to environmental conditions such as moisture availability, light exposure, substrate composition, and other external factors [65,66,67]. Furthermore, although CAP treatments were applied under standardized conditions and seeds were exposed in a single layer to ensure uniform treatment delivery, intrinsic morphological differences among individual seeds may have affected the extent to which the treatment influenced their biological responses [37,68].

Nevertheless, despite the considerable biological variability observed in the datasets, as reflected by the relatively high coefficients of variation, the treatment effects remained statistically significant and exhibited substantial effect sizes. These findings indicate that the observed responses cannot be attributed solely to random variation, but rather reflect a genuine biological effect associated with CAP exposure.

## 4. Materials and Methods

### 4.1. Cold Atmospheric Plasma Reactor Set-Up

**Figure 9:**
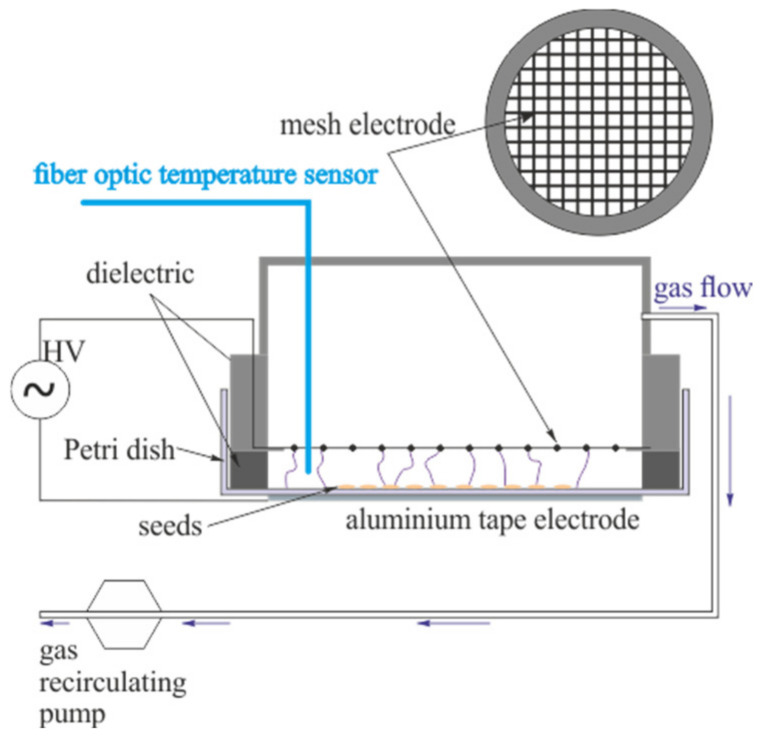
Cold atmospheric plasma by dielectric barrier discharge reactor (Faculty of Physics, Iasi Plasma Advanced Research Center (IPARC), Alexandru Ioan Cuza University of Iasi, Romania). Blue arrows indicate the direction of gas flow, while the different colors are used solely to distinguish the reactor components and do not represent quantitative or functional differences.

For this study, a CAP-DBD reactor configured for seed treatment under controlled conditions was used, the reactor set-up being presented in Figure 9 [69].

This set-up was powered by a sinusoidal alternating voltage (AC) source, with a value of 15.5 kV peak-to-peak and a frequency of 50 Hz. The electrode configuration consisted of an upper electrode of a metal mesh type (grid electrode) and a lower electrode made of aluminum strip, between which a discharge gap of 3 mm was maintained. The dielectric material used was glass, with a diameter of 100 mm.

### 4.2. Cold Atmospheric Plasma Treatment

A predetermined number of seeds (100 seeds) were evenly placed on the bottom of a glass Petri dish. The Petri dish served as support and was positioned on the aluminum strip electrode (ground electrode). Then, the Petri dish with seeds was inserted into the reactor, under the mesh electrode. The high voltage (HV) source was turned on to generate cold plasma in the discharge space (3 mm gap). Thus, the seeds were subjected to atmospheric cold plasma treatment for 1 min, 2 min, 3 min and 4 min. A batch of untreated seeds (control) was used for comparison, and each treatment duration constituted a distinct experimental group for both types of tomato seeds (*Solanum lycopersicum* common variety and an IdB hybrid). After the expiration of the preset time (e.g., 1 min), the HV source (15.5 kV peak-to-peak) was turned off, and the seeds were removed from the reactor for use in subsequent experiments. The main objective of this procedure was to induce surface modifications (etching), chemical activation, or sterilization of the seeds by the action of reactive species generated by cold plasma at atmospheric pressure (electrons, ions, radicals, excited species), while maintaining a low-temperature regime (non-thermal treatment), as confirmed by the fiber optic sensor.

### 4.3. Experimental Design

#### 4.3.1. Seed Sowing

In the experiment carried out in 2025, 100 seeds were planted for each of the two chosen tomatoes varieties, as follows:

- 20 control samples
- 20 samples treated with plasma for 1 min
- 20 samples treated with plasma for 2 min
- 20 samples treated with plasma for 3 min
- 20 samples treated with plasma for 4 min

The plants were cultivated in 4 alveolar trays of 60 cells each (10 × 6), with an approximate volume of 65 mL/cell. The cells were filled with a well-compacted peat substrate, approximately 2.5 cm thick, irrigated with 10 mL of water from the city’s public distribution network (Timișoara, Romania). In each cell, 2 seeds were placed at approximately 2 cm, over which a thin layer of loosened soil was added and irrigated with another 5 mL of water/cell. Following planting, for 47 days, indirect irrigation of the plants was carried out with a volume of 250 mL of water/drainage tray, once every 2 days, the volume being adjusted depending on the degree of soil dryness.

#### 4.3.2. Growth and Development of Seedlings

During the 47 days, the growth and development of the seedlings was observed, and the germination rate was determined (GR = Number of sprouted seeds/Total number of seeds sown × 100, %).

At the end of the growth period, each seedling was weighed using a precision analytical balance to determine the fresh biomass. The seedlings were then dried in an oven for 90 min at a temperature of 90 °C and weighed to determine the dry biomass. Subsequently, they were placed in porcelain crucibles and incinerated in the calcination furnace for 120 min at 500 °C. The resulting ash was weighed to determine the mass of accumulated inorganic substances.

### 4.4. Data Analysis

All statistical analyses were performed using R software (version 4.6.0) for data preprocessing and normality testing (Shapiro–Wilk test), while additional statistical tests, including one-way ANOVA, Levene’s test for homogeneity of variances, and Tukey’s HSD post-hoc analysis, were carried out using PAST software (version 4.03) and R statistical software. Graphical representations, including violin plots, were generated using GraphPad Prism (version 11.0.2).

Descriptive statistical parameters for the gravimetric data (minimum, maximum, mean, standard deviation, coefficient of variation, skewness, kurtosis and confidence intervals) were calculated using Microsoft Excel (Microsoft Office 2016). These parameters were used to characterize the central tendency, dispersion, and distribution shape of the experimental data prior to inferential statistical analysis. Violin plot representations were utilized to characterize the distribution shape, value density, degree of dispersion, and potential asymmetries in each data sets.

The statistical analysis employed a series of parametric tests in order to evaluate the effects of CAP treatment on the investigated biological parameters. Data normality was assessed using the Shapiro–Wilk test, while homogeneity of variances was evaluated with Levene’s test. Given the substantial variability observed within experimental groups and the unequal variances detected in several datasets, differences among treatment groups were analyzed using Welch’s one-way analysis of variance (Welch ANOVA). Statistical significance was established at *p* < 0.05. Post-hoc pairwise comparisons were conducted using Tukey’s test to identify specific group differences, following a positive ANOVA result. In cases where pairwise differences remained difficult to detect due to high data variability, the Dunnett T3 multiple comparison test was additionally employed to further explore treatment effects. To evaluate the influence of both genotype and treatment duration, as well as their potential interaction, a two-way ANOVA was performed, allowing the assessment of the relative contribution of genetic background and CAP exposure time to the observed biological responses.

## 5. Conclusions

Cold atmospheric plasma (CAP) treatment significantly influences the early development of *Solanum lycopersicum* seedlings, the magnitude and direction of the response being strongly dependent on exposure duration. Across both investigated genotypes, a treatment duration of 1 min consistently produced the most favorable outcomes out of the four exposure times evaluated, resulting in increased fresh, dry, and mineral biomasses compared to longer exposure times. In contrast, prolonged treatments generally reduced growth-related parameters, suggesting the existence of a narrow exposure window in which CAP acts as a beneficial priming stimulus, whereas excessive exposure may induce physiological stress and limit plant development.

The statistical analyses confirmed that treatment duration was the primary factor driving the observed responses, with significant differences detected for all gravimetric parameters and moderate to large effect sizes. Although the IdB hybrid exhibited a more pronounced positive response to the 1 min treatment than the common tomato variety, the two-way ANOVA did not reveal a significant genotype effect or genotype-treatment interaction, indicating that, under the conditions tested, CAP exposure time exerts a greater influence on early seedling growth than genetic background.

The present results support the potential use of CAP as a sustainable, non-chemical seed-priming technology capable of enhancing early plant development while aligning with current agricultural strategies aimed at reducing dependence on synthetic inputs. The identification of an optimal treatment duration further highlights the importance of precise process control when implementing plasma-based technologies in crop production systems.

Nevertheless, given the controlled laboratory conditions under which the present study was conducted, the singular focus on early developmental stages of the two tomato genotypes, the relatively high biological variability observed within some experimental groups, together with differences in germination success and sample size among treatments, the precision of certain estimates could be biased. Furthermore, only morphological and gravimetric parameters were evaluated, while the physiological, biochemical, and molecular mechanisms underlying the observed responses were not directly investigated.

Taking these aspects into consideration, future studies should therefore include a broader range of genotypes, larger sample sizes, longer cultivation periods, and greenhouse or field-scale validation experiments. Moreover, the integration of physiological, biochemical, and gene-expression analyses would provide a more comprehensive understanding of the mechanisms through which CAP influences seed germination and plant development, in order to optimize treatment protocols and facilitate the practical implementation of plasma technologies in sustainable agriculture.

## Figures and Tables

**Table 1:** Descriptive statistics for gravimetric parameters.

		Exposure Time	Min.	Max.	Mean	Str. Dev.	Coef. Var.	Skewness	Kurtosis	CI_95%_
*Solanum lycopersicum* common variety	Fresh biomass	1 min	0.136	0.434	0.257	0.096	0.375	0.668	0.350	[0.1774, 0.3356]
		2 min	0.007	0.649	0.217	0.202	0.903	1.343	1.736	[0.0852, 0.3491]
		3 min	0.047	0.135	0.086	0.035	0.413	0.301	−1.440	[0.0575, 0.1141]
		4 min	0.013	0.132	0.069	0.037	0.541	−0.214	0.349	[0.0445, 0.0931]
		control	0.026	0.356	0.146	0.096	0.659	1.249	1.500	[0.0863, 0.2056]
	Dry biomass	1 min	0.012	0.027	0.017	0.006	0.348	0.355	−0.175	[0.0128, 0.0210]
		2 min	0.004	0.041	0.014	0.012	0.862	1.364	1.809	[0.0063, 0.0226]
		3 min	0.006	0.009	0.007	0.010	0.202	0.507	−1.785	[0.0056, 0.0078]
		4 min	0.003	0.009	0.006	0.003	0.432	−0.533	−0.114	[0.0043, 0.0076]
		control	0.005	0.023	0.010	0.006	0.596	0.824	0.462	[0.0066, 0.0144]
	Ash content	1 min	0.003	0.010	0.005	0.002	0.411	1.507	3.265	[0.0036, 0.0065]
		2 min	2 × 10^−4^	0.017	0.004	0.005	1.281	2.089	4.921	[0.0012, 0.0077]
		3 min	0.001	0.002	0.001	4.8 × 10^−4^	0.388	0.885	2.636	[0.0009, 0.0016]
		4 min	0.001	0.003	0.002	0.001	0.506	0.079	−1.225	[0.0013, 0.0025]
		control	0.001	0.006	0.003	0.002	0.568	−0.022	−1.355	[0.0020, 0.0042]
*Solanum lycopersicum* IdB hybrid	Fresh biomass	1 min	0.075	1.041	0.419	0. 313	0.745	1.076	0.294	[0.0786, 0.1496]
		2 min	0.014	0.124	0.064	0.051	0.796	0.328	−3.159	[0.0540, 0.1224]
		3 min	0.020	0.227	0.082	0.060	0.724	1.323	1.997	[0.0487, 0.1162]
		4 min	0.012	0.184	0.088	0.052	0.594	0.381	0.106	[0.0540, 0.1224]
		control	0.014	0.232	0.114	0.065	0.572	−0.007	−0.563	[0.0786, 0.1496]
	Dry biomass	1 min	0.005	0.060	0.025	0.017	0.710	1.141	0.551	[0.0143, 0.0349]
		2 min	0.001	0.009	0.005	0.003	0.703	0.977	1.398	[0.0015, 0.0080]
		3 min	0.002	0.014	0.006	0.004	0.561	1.202	1.370	[0.0043, 0.0083]
		4 min	0.002	0.013	0.007	0.003	0.515	0.668	0.448	[0.0044, 0.0088]
		control	0.002	0.015	0.004	0.008	0.514	0.093	−0.886	[0.0061, 0.0108]
	Ash content	1 min	1.1 × 10^−3^	1.7 × 10^−2^	7.2 × 10^−3^	5.3 × 10^−3^	0.735	0.982	0.210	[0.0041, 0.0104]
		2 min	3.0 × 10^−4^	2.8 × 10^−3^	1.3 × 10^−3^	1.1 × 10^−3^	0.816	1.048	0.919	[0.0003, 0.0024]
		3 min	8.0 × 10^−4^	5.9 × 10^−3^	2 × 10^−3^	1.4 × 10^−3^	0.705	2.245	6.186	[0.0012, 0.0028]
		4 min	4.0 × 10^−4^	3.6 × 10^−3^	1.7 × 10^−3^	1.1 × 10^−3^	0.641	0.200	−0.793	[0.0011, 0.0025]
		control	6.0 × 10^−4^	6.1 × 10^−3^	2.5 × 10^−3^	1.4 × 10^−3^	0.568	0.998	2.015	[0.0018, 0.0033]

**Table 2:** ANOVA and Welch’s Test results for gravimetric parameters of *Solanum lycopersicum* common variety.

	ANOVA Test Results			Welch’s Test Results		
	F	*p*	Omega^2^	F	df	*p*
Fresh biomass	3.933	0.0095	0.2225	7.607	17.07	0.001041
Dry biomass	3.524	0.0158	0.1976	7.07	17.35	0.00145
Ash content	2.753	0.0423	0.143	7.942	17.89	0.0007271

Note: F represents the test statistic, df represents the degrees of freedom, *p* represents the probability value and omega squared represents the effect size. For *p* values < 0.05, data are considered statistically significant. The significance levels considered for the effect size are small effect (0.01–0.06), moderate effect (0.06–0.14), large effect (>0.14).

**Table 3:** ANOVA and Welch’s Test results for gravimetric parameters of *Solanum lycopersicum* IdB hybrid.

	ANOVA Test Results			Welch’s Test Results		
	F	*p*	Omega^2^	F	df	*p*
Fresh biomass	9.233	1.66 × 10^−5^	0.4019	3.349	16	0.03579
Dry biomass	8.447	3.80 × 10^−5^	0.3781	3.325	15.85	0.03691
Ash content	7.518	0.0001049	0.3473	3.168	16.18	0.04229

Note: F represents the test statistic, df represents the degrees of freedom, *p* represents the probability value and omega squared represents the effect size. For *p* values < 0.05, data are considered statistically significant. The significance levels considered for the effect size are small effect (0.01–0.06), moderate effect (0.06–0.14), large effect (>0.14).

**Table 4:** Two-way ANOVA Test results for genotype and CAP exposure times interaction.

		Fresh Biomass		Dry Biomass		Mineral Biomass	
	df	F	*p*	F	*p*	F	*p*
Genotype:	1	0.09205	0.7624	0.01056	0.9184	0.0001258	0.9911
Treatment time:	4	11.99	1.056 × 10^−7^	10.76	4.824 × 10^−7^	8.665	7.131 × 10^−6^
Interaction:	4	2.46	0.05185	2.084	0.09045	1.763	0.1443

Note: F represents the test statistic, df represents the degrees of freedom and *p* represents the probability value. For *p* values < 0.05, data are considered statistically significant.

## Data Availability

The raw data supporting the conclusions of this article will be made available by the authors on request.
